# Canada

**Published:** 1867-08

**Authors:** 


### *Canada.*

We have received a circular from Dr. Russel, Secretary of the Quebec Medical Society, looking toward the organization of the profession throughout the "Dominion of Canada," under the style of the "Canadian Medical Association." The Quebec Society adopted the subjoined resolutions:

*Resolved, 1.* That in the interest of the public, and the medical profession, it is desirable to adopt such means as will insure an *uniform* system of granting license to practice Medicine, Surgery, and Midwifery, throughout the Dominion of Canada.

*Resolved, 2.* That in future, all medical degrees or diplomas, of Universities, Colleges, or Schools of Medicine, shall have merely an honorary value, and licenses to practice Medicine, Surgery, or Midwifery, in the Dominion of Canada, shall be granted by a Central Board of Examiners, in each Province, before whom all holders of Degrees in Medicine, or Diplomas for Surgery, or Midwifery, shall appear for examination.

*Resolved, 3.* That a committee of seven members be named by the Medical Society, to confer with the various Universities, Colleges, and Medical Schools in Canada, on the subject of the establishment of a Central Board of Examiners, before which, all candidates for license to practice medicine in the Dominion of Canada, shall be examined.

*Resolved, 4.* That the Quebec Medical Society recommends the calling of a Convention of Medical Delegates, from Universities, Colleges, Schools, Medical Societies, etc., in the Dominion of Canada; to meet at the city of Quebec, on the second Wednesday in October, 1867, for the purpose of adopting some concerted action, on the subject of medical legislation, in conformity with this report, and for the formation of a "Canadian Medical Association."

We trust our brethren in Canada will succeed in organizing their efforts to the better advancement of medical science; but their "Central Board of Examiners will prove a failure. The same idea is a favorite one in this region, also—one enthusiastic individual, indeed, having tried to commit the Illinois Medical Society to its support before the Legislature. "*Who shall guard the Shepherds?*" Whatever legislatures touch they mar. The frogs "missed their figure" in getting a king. Let the medical profession say to politicians, "Hands off!" Let it ask only a clear field and no favors. Neither ask nor give quarter in its contests. In Society the gentleman always takes his appropriate position. There is a higher law which regulates these matters than can be enacted by the political bawds who throng the capitols.

